# Differential diagnosis between Parkinson's disease and essential tremor using the smartphone's accelerometer

**DOI:** 10.1371/journal.pone.0183843

**Published:** 2017-08-25

**Authors:** Sergi Barrantes, Antonio J. Sánchez Egea, Hernán A. González Rojas, Maria J. Martí, Yaroslau Compta, Francesc Valldeoriola, Ester Simo Mezquita, Eduard Tolosa, Josep Valls-Solè

**Affiliations:** 1 School of Medicine, University of Barcelona (UB). Barcelona, Catalonia, Spain; 2 Mechanical Engineering Department (EPSEVG). Politechnical University of Catalonia (UPC). Barcelona, Spain; 3 Parkinson’s Disease & Movement disorder unit. Neurology department. Hospital Clínic / IDIBAPS. CIBERNED Barcelona, Catalonia, Spain; 4 Mathematica Department (EPSEVG). Politechnical University of Catalonia (UPC). Barcelona, Spain; 5 EMG and Motor Control Unit. Neurology department. Hospital Clínic of Barcelona. Barcelona, Spain; University of Toronto, CANADA

## Abstract

**Background:**

The differential diagnosis between patients with essential tremor (ET) and those with Parkinson’s disease (PD) whose main manifestation is tremor may be difficult unless using complex neuroimaging techniques such as ^123^I-FP-CIT SPECT. We considered that using smartphone’s accelerometer to stablish a diagnostic test based on time-frequency differences between PD an ET could support the clinical diagnosis.

**Methods:**

The study was carried out in 17 patients with PD, 16 patients with ET, 12 healthy volunteers and 7 patients with tremor of undecided diagnosis (TUD), who were re-evaluated one year after the first visit to reach the definite diagnosis. The smartphone was placed over the hand dorsum to record epochs of 30 s at rest and 30 s during arm stretching. We generated frequency power spectra and calculated receiver operating characteristics curves (ROC) curves of *total spectral power*, to establish a threshold to separate subjects with and without tremor. In patients with PD and ET, we found that the ROC curve of *relative energy* was the feature discriminating better between the two groups. This threshold was then used to classify the TUD patients.

**Results:**

We could correctly classify 49 out of 52 subjects in the category with/without tremor (97.96% sensitivity and 83.3% specificity) and 27 out of 32 patients in the category PD/ET (84.38% discrimination accuracy). Among TUD patients, 2 of 2 PD and 2 of 4 ET were correctly classified, and one patient having PD plus ET was classified as PD.

**Conclusions:**

Based on the analysis of smartphone accelerometer recordings, we found several kinematic features in the analysis of tremor that distinguished first between healthy subjects and patients and, ultimately, between PD and ET patients. The proposed method can give immediate results for the clinician to gain valuable information for the diagnosis of tremor. This can be useful in environments where more sophisticated diagnostic techniques are unavailable.

## Introduction

Parkinson’s disease (PD) and essential tremor (ET) are the most common tremor syndromes worldwide [1]. Differentiation between these two pathologies can sometimes be difficult. Approximately 20% of PD patients may be initially diagnosed as ET and vice versa and rates of misdiagnosis can be as high as 25% [2], even when managed by a movement disorder specialist. Particularly, the differentiation between the two diseases is more difficult at early stages, when however, a specific treatment would be particularly important [3]. Hereby, better diagnostic procedures have been developed to avoid incorrect treatment and delayed diagnosis. ^123^I-FP-CIT SPECT has proved to be the most powerful tool for the clinician in this matter, providing 97% sensitivity and 100% specificity for parkinsonism syndromes [1]. Nevertheless, it is a resource consuming test [4] and its use is limited to only 32 well developed countries worldwide [5]. Thus, cheaper non-invasive tests are needed that could be available in underdeveloped, less wealthy, countries.

Smartphone’s built-in accelerometers might have such potential if provided with a standardized recording and analysis systems. In fact, previous studies have shown that smartphone accelerometers are comparable to laboratory accelerometers in the assessment and classification of tremors [6–10], and although there is overlapping in the range of frequencies that PD and ET tremors exhibit, power spectrum analysis of accelerometer signals has proven to discriminate effectively between PD and ET [3,11–14]. Woods et al. [14] recently achieved discrimination between patients with PD and those with ET using smartphone accelerometers. However, their method could be difficult to implement in clinical routine given that it requires multiple manoeuvres, good training and specific equipment to perform distraction tests.

We examined whether the frequency spectrum analysis of the signal recorded at rest and posture with a smartphone accelerometer could discriminate between PD and ET tremors under routine clinical conditions. Our system furnishes real time data that could contribute to establish a more accurate clinical assessment of patients with tremor [15] and is, therefore, applicable to their diagnosis.

## Methods

This prospective tremor analysis study was carried on in healthy subjects and in patients selected consecutively after their follow-up visit at the Movement Disorder Unit of the Hospital Clinic of Barcelona between October 2015 and December 2016. All measurements were undertaken in less than 3 minutes. In patients, this was done *in situ*, after a routine follow-up visit with the movement disorder specialist.

### Participants

We included patients that presented with visually evident hand tremor and the established diagnosis or strong diagnostic suspicion of PD or ET. Most patients followed the Movement Disorders Society criteria for ET [16] or the UK Brain Bank criteria for PD [17]. In cases fulfilling incomplete criteria, the diagnosis was based on expert opinion or was left open until a follow-up evaluation. Intensity of tremor was evaluated with the Fahn-Tolosa-Marín scale [18] for the ET and the UPDRS for tremor in patients with Parkinson’s disease and we selected only patients with mild tremor, with scores of 1 or 2 in both scales. We excluded patients with a known neurological disease other than PD or ET or those who, having been diagnosed of PD or ET, showed signs of other conditions indicating either peripheral or central nervous system disorders, or those who had any neurological or mechanical impairment that could prevent the recording. Pharmacological or surgical treatment of tremor were not exclusion factors. We also included in the study patients with tremor of undecided diagnosis (TUD). These were typically patients who were interviewed for the first time and they could not be correctly classified because they presented either combined features of PD and ET or equivocal signs. Their recordings were separated and stored for further analysis to determine if the developed test could classify them correctly once their diagnoses were established in follow-up clinical evaluation with the support of complementary tests, such as ^123^I-FP-CIT SPECT electromyography (EMG) or others. The control group (CG) consisted of subjects without any known neurological disorder, specifically tremor. To this end all these CG subjects underwent a comprehensive neurological physical examination. Family history of tremor and intake of tremor inducing medication [19] were exclusion criteria.

After the pre-selection, patients and healthy subjects were informed of the procedure and asked to sign a consent form. The study was approved by the Ethics Committee of Hospital Clinic of Barcelona in accordance with the ethical standards laid down in the Declaration of Helsinki and its later amendments.

### Procedure

Tremor was recorded using the in-built triaxial accelerometer of an iPhone 5S (Apple Inc, USA) with a sampling rate of 100 Hz. Data were processed by *SensorLog* application software [20], and sent online to a remote computer for further analysis.

**Fig 1:**
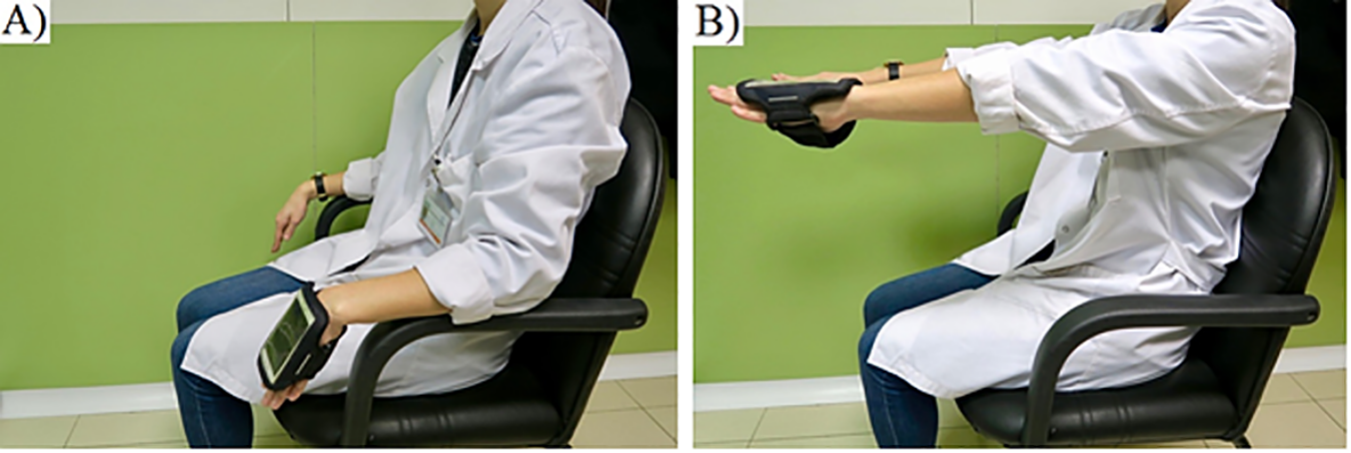
1A, Recording tremor in rest position. 1B, Recording tremor in postural position.

Subjects were sitting on a comfortable chair with armrests. The smartphone was placed on the dorsum of the most affected hand in patients or on the dominant hand in CG subjects. The device was attached to the hand using a common running armband. Recordings were taken during 30 seconds in two conditions: At rest (condition ‘Rest’), while subjects were resting their forearm on top of the armrest as relaxed as possible, with hands hanging from the edge of the armrest, as shown in Fig 1A, and with the arms stretched (condition ‘Posture’), while subjects maintained both upper limbs fully extended in front of them, with the palms facing the ground, as shown in Fig 1B.

### Data analysis

Data were imported and processed using Matlab v. R2010a (Mathworks Inc., USA).The magnitude of signal from the accelerometer, which is the square root of the sum of the squares of each acceleration component, was pre-processed, by removing drifts and gravitational acceleration components over time, and properly trimmed (0.5 s in both sides), to avoid side effects from manually switching on and off the recording. The codes are reported as a supplementary material (S1 File). Power spectral density was calculated by using the Welch periodogram [21]. We ran an average at every segment of 3 s of signal recording with 50% overlapping of the Hanning windows. Researchers analysing the data (AJSE and HAGR) were blinded to the diagnosis.

**Fig 2:**
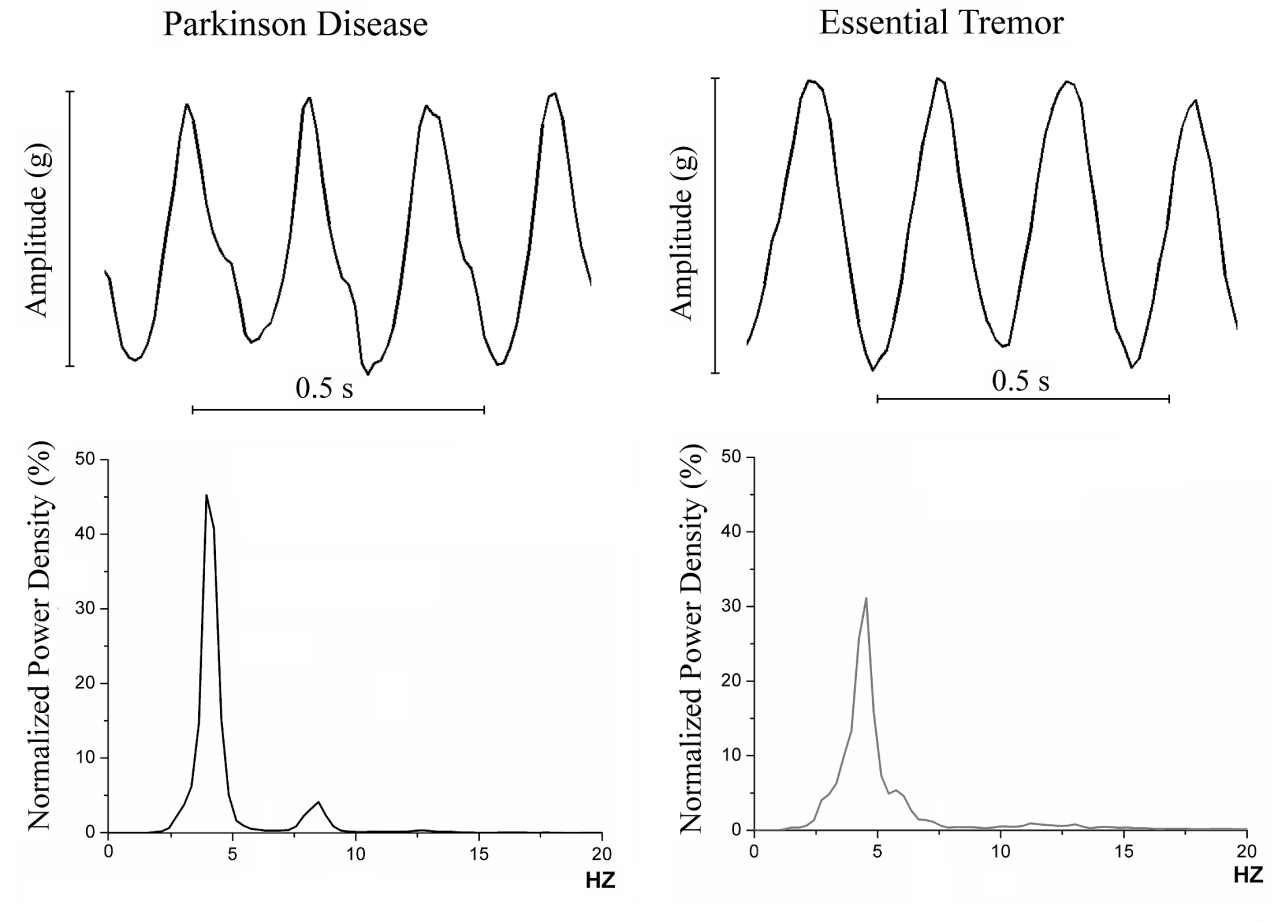
Upper section, examples of the smartphone’s accelerometer signal morphology in Parkinson’s Disease (PD) and essential tremor (ET). Lower section, examples of normalized power spectral density of tremor in PD and ET subjects.

We first obtained the power spectrum and calculated the total spectral power in healthy subjects and in PD and ET patients, lumped together as a single group, to establish a threshold separation between clinically relevant tremor (PD and ET) and physiological tremor. Examples of PD and ET tremor recordings and their respective spectral power are represented in Fig 2. Total spectral power is defined as the area under the curve of the power spectrum and it is a reliable tremor amplitude measure [22,23]. Later, the two groups were compared using ROC curves in rest and posture and determined the cut-off value in which the highest discrimination between the two groups was obtained.

As patients do not necessarily present relevant tremor in both positions, we considered pathological those with measures above the threshold in at least one of the two positions. Patients who were not meeting this criterion in the first part of the study were excluded for the second part, as they would have been considered physiological by the smartphone in an hypothetical automated procedure.

After this first analysis, the following features were computed from power spectral analysis in PD and ET patients:

*Median power frequency*. Represents the frequency where 50% of the power lies below it and the remaining 50% lies above it.

*Power dispersion*. Represents the width of a frequency band containing 90% of total power; centred at the median power frequency.

*Peak power frequency*. Represents the frequency where the maximum power was observed.

*Harmonic index*. Represents the quotient between the area under the curve of the power spectral density and a rectangle bounded on the sides by the frequency band of interest (0–20 Hz) and vertically from 0 to the height of the highest peak of amplitude. The harmonic index is the proportion of the area of this rectangle lying above the power spectrum itself [6].

Two new discriminatory features were applied:

*Relative Power Contribution to the first harmonic (RPC)*: It is based on the idea that PD and ET tremors may differ in relation of the proportional contribution of the main frequency peak to the total spectral power, because of their differences in relation with the frequency harmonics that can appear in these pathologies [6,11,12,24]. RPC is calculated from the quotient between the power spectral density of harmonics within the frequency range of *f*_1_ (threshold) and 20 Hz and the total normalized power spectral density for a frequency range of 0 to 20 Hz. The equation of RPC can be written as follows:

$$RPC=\frac{\int_{f_{1}}^{20}S\;(f)\cdot df}{\int_{0}^{20}S\;(f)\cdot df}$$

Where *S*(*f*) is the normalized power spectral density and *f*_1_ is the threshold to divide the harmonics from the fundamental frequency, which is defined by enclosing the 95% of the normalized power spectral density of the first peak.

*Relative Energy (RE)*. It is based on the assumption that the relation between total spectral power in rest and posture should be higher in PD patients than in ET patients, given the clinical features of the diseases. Theoretically, PD patients should present higher amplitudes of rest tremor (position A) than postural tremor (position B), and the opposite way for ET patients. Therefore, RE is calculated from the quotient between the total normalized power spectral densities in rest and in posture (respectively, A and B in the formula) for a frequency range of 0 to 20 Hz. The equation of RE can be written as follows:

$$RE=\frac{\int_{0}^{20}S_{A}\;(f)\cdot df}{\int_{0}^{20}S_{B}\;(f)\cdot df}$$

Where *S*_*A*_(*f*) is the normalized power spectral density in rest and *S*_*B*_(*f*) is the normalized power spectral density in Posture.

### Statistical analysis

We first examined the distribution of signal data with the Anderson Darling test. For comparison of non-linear distributed data, we used the non-parametric Mann-Whitney U test. This was used to compare the median value of all examined features, first between healthy subjects and patients with tremor and, then, between ET and PD patients. Only a p-value equal or lower than 0.05 was considered significant.

Once the discriminative features were found, we performed a natural logarithmic transformation of non-linear discriminative data to obtain its normal distribution and examine the receiving operating characteristics (ROC) curves. The efficiency of the discrimination of variables between PD and ET patients was evaluated using the area under the ROC curve (ROC-AUC). This parameter measured the probability of correct discrimination for one given subject. The feature that presented higher discrimination values according to the ROC-AUC was used to establish a threshold to separate ET from PD patients.

Sensitivity and specificity of the optimal cut-off value of ROC was calculated for the test. All statistical analyses were done using SPSS v. 23 (IBM Inc., NYC, USA). The level of significance was set at 95% for all the tests.

## Results

The subjects initially recruited were 54: 17 PD patients, 17 ET patients, 7 TUD patients and 13 healthy subjects. However, data from one of the ET patients initially recruited had to be excluded from the analysis because of a high frequency artefact that contaminated the signal and data in one healthy subject were not fit for analysis because of software incompatibility with an earlier version of the mobile application. All PD patients and 11 patients with ET were on medication at the time of recording and 2 patients with ET had undergone deep brain stimulation surgery (none in the PD group). Table 1 shows the main demographic characteristics of the 40 patients and 12 healthy subjects, which data were analysed. Table 2 describes in detail tremor and other individual characteristics of the TUD patients.

**Table 1:** Demographic characteristics of the subjects studied (n = 52).

	Parkinson’s Disease (n = 17)	Essential Tremor (n = 16)	Control Group (n = 12)	Tremor of Undecided Diagnosis (n = 7)
Female; number (%)	5 (29.4)	11 (68.8)	6 (50.0)	4 (57.1)
Male; number (%)	12 (70.6)	5 (31.2)	6 (50.0)	3 (42.9)
Mean age; years (SD, range)	71.3 (10.9, 48–91)	73.8 (12.4, 39–89)	69.0 (8.6, 53–81)	67.6 (19.6, 25–84)
Mean time since diagnosis; years (SD, range)	8.9 (5.2, 2–22)	14.1 (9.0, 2–30)	NA	NA

**Table 2:** Patients with tremor of undecided diagnosis (TUD).

Patient	Sex	Age	Family history	Clinical features	^123^I-FP-CIT SPECT	1 year clinical history re-evaluation	Final diagnosis
TUD01	Male	64	Daughter: ET	Bilateral postural and rest tremor with rigidity and bradikinesia for 8 years. Patient was initially misdiagnosed as ET and reclassified as PD one year later after ^123^I-FP-CIT SPECT results.	Positive	No changes	Misdiagnosed as ET. Final diagnosis: PD
TUD02	Female	75	Sister: PD Mother: ET	Left unilateral postural tremor. Positive family history for ET and PD. No response to propranolol. ^123^I-FP-CIT SPECT is ordered.	Negative	Negative SPECT. Patient is diagnosed of ET	Atypical ET
TUD03	Female	25	Mother: ET Grandfather: ET Brother: ET	Postural and kinetic mild tremor with poor response to low dose propranolol treatment. EMG is ordered.	None	EMG results: Intentional high frequency tremor compatible with enhanced physiological tremor	Enhanced physiological tremor
TUD04	Male	76	None	14 years history of unilateral high amplitude, low frequency rest tremor, and a slight component of cephalic tremor, without signs of parkinsonism. He was treated with levo dopa without response, and performed ^123^I-FP-CIT SPECT, with negative results. Given the high level of suspicion, he underwent a second SPECT.	Negative x2	No changes	Atypical ET
TUD05	Female	84	None	Bilateral rest tremor and cephalic tremor for 14 years. She underwent ^123^I-FP-CIT SPECT 12 years ago with negative results. No response to propranolol.	Negative	No changes	Atypical ET
TUD06	Female	74	Father: ET	9 years history of bilateral rest and postural tremor and patient was diagnosed of ET with good response to propranolol. After 5 years, patient starts with bradikinesia and rigidity. ^123^I-FP-CIT SPECT is performed with abnormal results and was diagnosed of PD as well, with good response to treatment.	Positive	No changes	PD and ET concomitance
TUD07	Male	75	None	Patient with unilateral rest and postural tremor for 6 months. Mild bradikinesia. Poor tolerance to levodopa.	None	Tremor remained unaltered. Bradikinesia and rigidity were more evident.	PD

TUD, Tremor of undecided origin. PD, Parkinson’s disease. ET, essential tremor. EMG, Electromyography.

### Part 1: Discrimination between patients with tremor and healthy subjects

**Fig 3:**
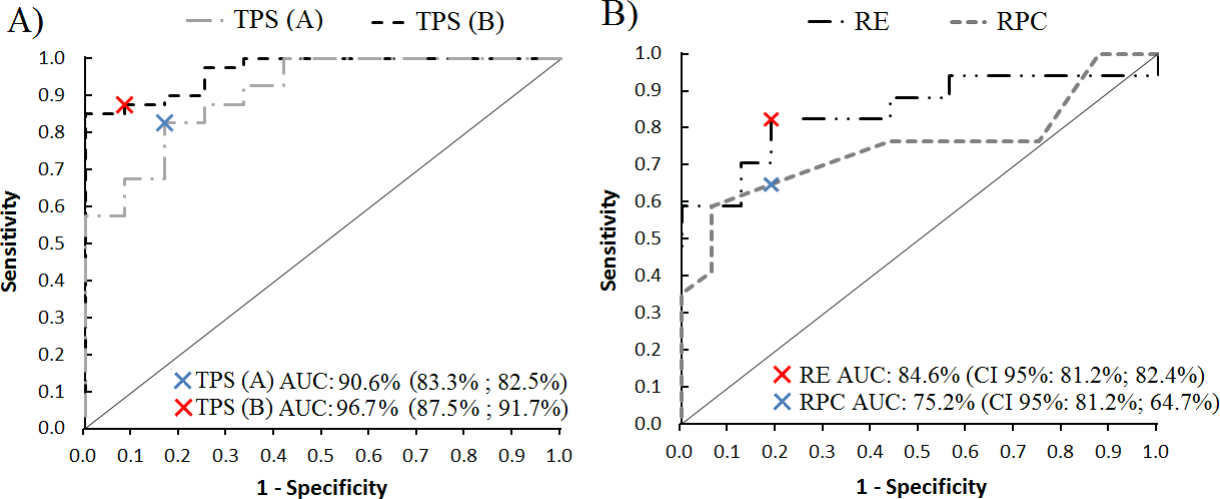
3A, ROC curves for rest total power spectra (TPS A) and postural total power spectra (TPS B) comparing tremulous and healthy subjects. 3B, ROC curves for relative energy feature (RE) and Relative Power Contribution to the first harmonic feature (RPC). Blue and red crosses mark the highest discriminative threshold for each ROC curve. AUC, area under the curve. CI, confidence interval, followed by sensitivity and specificity for the given value.

Total spectral power in Rest significantly differed (p-value <0.001) between patients (median 0.3 mg^2^·s, Interquantile Range (IQR) 0.09–4.2) and healthy subjects (median 0.027 mg^2^·s, IQR 0.017–0.057). Total spectral power in Posture significantly differed (p-value <0.001) between patients with tremor (median 1.4 mg^2^·s, IQR 0.09–6.5) and healthy subjects (median 0.1 mg^2^·s, IQR 0.08–0.2). ROC curves of Rest and Posture are depicted in Fig 3A. ROC-AUC was 90.6% (95% confidence interval: 86.0–95.2) for Rest and 96.7% (94.5–98.9) for Posture. For Rest, the cut-off value was set at 0.074 mg^2^·s, according to highest discrimination point (sensitivity 83.3%, specificity 82.5%). For Posture, the cut-off value was set at 0.35 mg^2^·s (sensitivity 87.5%, specificity 91.7%). For Rest, 33 out of 40 tremulous patients were classified as having pathological tremor and 10 out of 12 healthy subjects were classified as having physiological tremor. For Posture, 36 out of 40 tremulous patients were classified as having pathological tremor and 11 out of 12 healthy subjects were classified as having physiological tremor. The combination of the two conditions, taking into account subjects that presented total power spectral values above the threshold in at least one of the recordings, showed positive identification of tremor in 39 out of 40 patients and in 2 out of 12 healthy subjects (97.96% sensitivity and 83.3% specificity).

### Part 2: Discrimination between PD and ET

17 out of 17 PD and 15 out of 16 ET patients obtained positive identification of tremor in part 1. Thus, one ET patient was excluded from the analysis, and 32 were finally included in part 2. Tremor characteristics of both groups are shown in Table 2. PD and ET patients did not differ in peak power frequency, median power frequency, power dispersion, harmonic index or relative power contribution in rest (p-values >0.05 in all comparisons). However, significant differences were found for the analysis of RPC in Posture, with median values of 0.25, IQR 0.15–0.41 for PD and 0.10, IQR 0.08–0.11 for ET (p = 0.014) and in the analysis of RE with median values of 1.88, IQR 0.56–3.94 for PD and 0.03, IQR 0.02–0.15 for ET (p <0.001).

**Fig 4:**
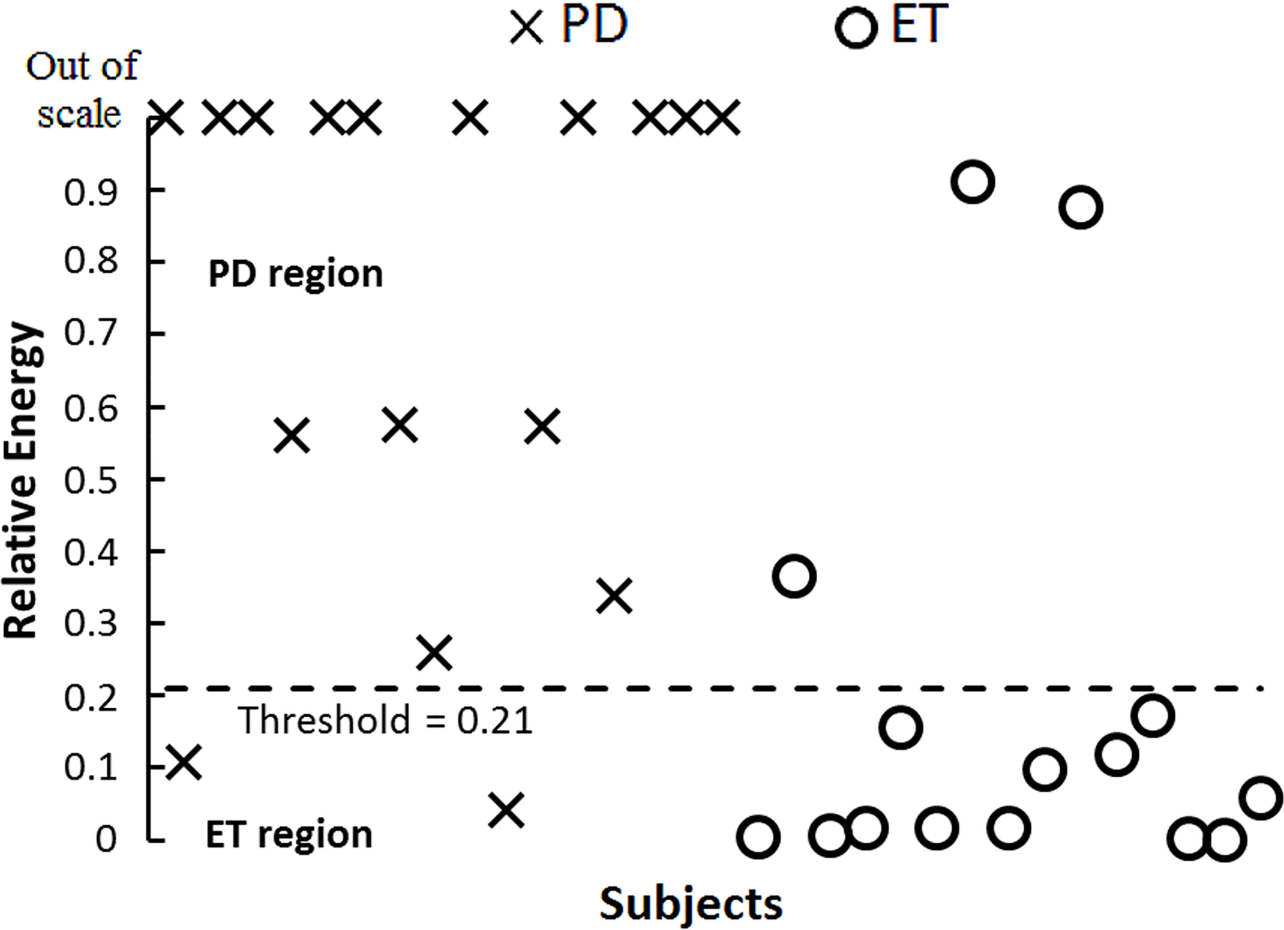
Discrimination of Parkinson’s disease and essential tremor subjects using the threshold obtained from relative energy feature ROC curve. 15 of 17 Parkinson’s and 13 of 15 essential tremor patients were correctly classified. PD region, values in this region are classified as Parkinson’s disease. ET region, values in this region are classified as essential tremor.

From the ROC-AUC values for RPC in posture, the highest discriminative point was set at 0.125 (74.9%, CI 95% 66.0–83.8). For RE, the highest discriminative point was set at 0.21 (89.8%, 84.5–94.8), as shown in Fig 3B. Graphic representation of RE values using the established threshold is shown in Fig 4, where 27 out of 32 patients were classified correctly (84.38% discrimination accuracy).

**Fig 5:**
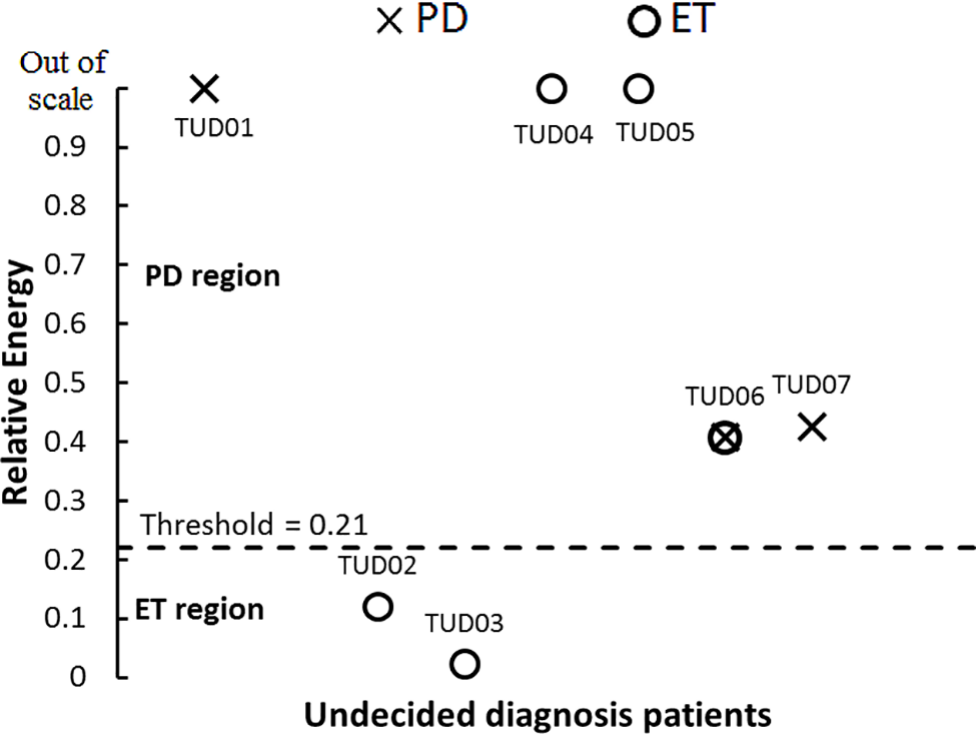
Relative energy threshold was tested with patients with undecided diagnosis at the moment of the recording. 2 out of 2 Parkinson’s disease and 2 out of 4 essential tremor patients were correctly classified. One subject that presented Parkinson’s and essential tremor concomitance was classified as Parkinson’s disease by the test. PD, Parkinson’s disease ET, essential tremor. AP, atypical presentation.

We reviewed the clinical histories of TUD patients one year after the recordings to confirm their final diagnosis. Two patients received the diagnosis of PD, 4 received the diagnosis of ET and one was diagnosed as having PD plus ET concomitance. TUD patient’s characteristics are shown in Table 3. Using the threshold values established in our analysis, the test classified correctly both PD patients, 2 of the 4 ET patients and the patient having PD plus ET was classified as PD, as depicted in Fig 5.

**Table 3:** Kinematic features of the spectral power analysis for rest and postural tremor in Parkinson’s disease and essential tremor subjects.

Parameter	Position	Parkinson’s disease		Essential tremor		p-value*
		*Median*	*IQR*	*Median*	*IQR*	
*Median power frequency*	A	4.90	4.09–5.30	5.15	4.40–5.50	0.4167
	B	5.50	4.69–5.79	5.70	5.29–6.01	0.2870
*Power dispersion*	A	1.40	0.98–2.13	3.40	1.21–4.01	0.1542
	B	3.90	2.32–5.72	2.85	0.90–4.12	0.1652
*Peak power frequency*	A	5.00	3.98–5.28	5.15	4.29–5.69	0.3174
	B	5.70	4.99–6.31	5.85	5.31–6.00	0.3014
*Harmonic index*	A	0.04	0.02–0.03	0.07	0.04–0.08	0.0532
	B	0.07	0.06–0.11	0.05	0.03–0.05	0.0703
Relative Power Contribution**	A	0.13	0.10–0.19	0.11	0.08–0.17	0.664
	**B**	**0.25**	**0.15–0.41**	**0.10**	**0.08–0.11**	**0.0140**^**†**^
Relative Energy**	**A / B**	**1.88**	**0.56–3.94**	**0.03**	**0.02–0.15**	**0.0001**^**†**^

A, rest tremor. B, postural tremor. IQR, Interquartile range.

*Mann–Whitney U test is applied to compare the median between ET and PD groups.

** Self-developed kinematic features

† test is found to have statistic significance (p-value<0.05)

## Discussion

We have shown that the characterization of tremor and differentiation between tremor in PD and in ET is feasible with a smartphone accelerometer. We have also shown that this test could be performed in a few minutes and does not require sophisticated or expensive equipment and, therefore, can be used in clinical routine conditions. This is in contrast to other methods, such as the one proposed by Woods et al [14], which may reach better discrimination accuracy using a more complex and time-consuming test. We think that an appropriate balance between higher discriminatory values and feasibility in clinical routine conditions is required for the development of useful diagnostic tests.

Patients of our study were selected according to clinical diagnoses. Concerning the 2 PD and 3 ET patients who were misclassified, all five of them exhibited mild tremor and were on medication at the time of the recording. These are possible reasons for misclassification. The 2 PD and 3 ET patients who were misclassified had very mild tremor and were on medication at the time of recording. We think that the method used in our study might have a floor limitation in detection of low intensity tremor, when it reaches levels comparable to those of healthy subjects. In regards to the TUD group. our sample was too small to draw firm conclusions but 4 out of the 7 TUD patients had in common the presentation of tremor in both conditions of the study, as described in Table 2. These patients with atypical presentation of tremor may entail a difficult classification when the basis of discrimination between the two groups of patients is the comparison between Rest and Posture.

Concerning the possible impact of the weight of the smartphone (112 g) in our results, we think that it does not have a significant impact in the condition Rest, considering that in previous studies in healthy subjects under the same conditions (tremor recorded for 30 s in a chair with arm rests) the addition of 500 g and 1000 g weights did not change total spectral power with respect to the no-weight condition [22]. However, it has not been examined if the same applies also for the condition Posture and for PD and ET patients.

Although the diagnostic capability achieved in this study is remarkable, it is probably not superior to the one obtained with clinical examination alone. Nevertheless, it could offer support specially when tremor is mild and difficult to identify. In order to improve the accuracy of this method without implementing longer or more complex manoeuvers, we believe that studies on a larger number of patients are needed to define more accurately the thresholds used for classification. Additionally, the implementation of machine learning algorithms could be beneficial to describe new and more accurate discriminative features for the differential diagnosis not only of PD and ET, but also of other types of tremor. Moreover, our test was performed on patients with a clear history of PD or ET to avoid misclassification, but that means that they were also on treatment and sometimes tremor intensity was remarkably low. We think further studies should be performed in first visit patients consulting of tremor, to avoid medication or surgery suppression effects. It also could be of interest to analyse the gyroscope signal of the smartphone in search of more discriminative features.

The cut-off values described in this article have been developed for our own particular sample and their value should be checked in future studies. Moreover, other methods have been proposed, all of them with sample sizes comparable to ours. A large, multicentre study would be appropriate to establish better cut-off values and reach wide generalizability of the method. Some of the greatest advantages that smartphones can bring to the scientific community in the future are the direct online evaluation, which could be of use to those interested in physiological tremor suppression [25], and the fast communication of data between centres worldwide, via the application, for centralized analysis.

## Conclusion

We demonstrated the feasibility of a quick test using smartphones accelerometers to characterize tremor and recognize discriminatory features of tremor in PD and ET patients. The signal processing protocol could be implemented in a software application and give immediate results for the clinician to gain valuable information for the diagnosis of tremor. We report on a new accelerometric test derived from the relation between Rest and Posture total spectral power that, in combination with other existing features, can reach high discriminatory results between PD and ET tremors. We believe this method can become an efficient tool to help physicians to make diagnostic decisions, especially when other complementary tests are not available.

## Supporting information

S1 File MatLab instructions for data processing. (DOCX)
