# Supplementary material for: Differential diagnosis between Parkinson's disease and essential tremor using the smartphone's accelerometer
Source: PLoS One. 2017 Aug 25;12(8):e0183843. doi: 10.1371/journal.pone.0183843 (PMC5571972; doi:10.1371/journal.pone.0183843)
Supplement: S1 File — (DOCX) [file pone.0183843.s001.docx]

**Supplementary material**

S1 file: MatLab instructions for data processing.

%SIGNAL PRE-PROCESSING

%Load the signal of group A, patient 8 and hand position B

data=xlsread('GA08B.xlsx');

%Subtract non-desired signal from switch ON-OFF

data=data([175:3550],:);

%Subtract motion gravity to the acceleration signal

A(:,1)=data(:,1)-data(:,13);

A(:,2)=data(:,2)-data(:,14);

A(:,3)=data(:,3)-data(:,15);

%Gaussian filter to remove drift movements

h=fspecial('gaussian',[50 1],50);

A1=conv2(A,h,'same');

Af=A-A1;

%Subtract filter sided effects

Af=Af ([25:3350],:);

%Sampling rate

fs=100

%Welch filter with non-overlapping Hanning window of 3 seconds

[pxx,f]=pwelch(Af(:,1),[300],[],[300],fs);

[pyy,f]=pwelch(Af(:,2),[300],[],[300],fs);

[pzz,f]=pwelch(Af(:,3),[300],[],[300],fs);

%Save the results in txt file

P=[f,pxx,pyy,pzz,(pxx+pyy+pzz)./3];

save('accGA08B.txt', 'P','-ascii');

% Plot the power spectral density

figure(1)

hold on

plot(f,P(:,5),'linewidth',2)

xlabel('Frequency (Hz)','FontSize',14)

ylabel('Power (Ampitude)','FontSize',14)

title('PD08B','FontSize',16)

legend ('ax','ay','az','FontSize',14)

axis([0 20 0 inf])

grid on

hold off

%Plot the power spectral density in dB

figure(2)

hold on

plot(f,10*log10(P(:,5)),'linewidth',2)

xlabel('Frequency (Hz)','FontSize',14)

ylabel('Power (dB)','FontSize',14)

title('PD08B','FontSize',16)

legend ('ax','ay','az','FontSize',14)

axis([0 20 0 inf])

grid on

hold off

%KINEMATIC FEATURES ANALYSIS

FI = P(:,5);

Suma = 0;

for k =1:1:end

Suma = Suma+ P(k);

end

for k =1:1:end

PD = FI(k) ./ Suma;

end

%RELATIVE ENERGY FEATURE

RE = Suma;

% MEDIAN POWER FREQUENCY FEATURE

SumInt = 0;

%initial and end value of the analysis window.

Nin; NFin

for k = Nin:1: NFin

SumInt = SumInt + PD(k);

end

r =0;

repeat

k = k+1;

r = r + PD(k);

until r > SumInt/2;

end_repeat

PosMediana = k-1;

FMediana = FI(PosMediana);

%POWER DISPERSIÓN FEATURE

k = PosMediana;

Suma = 0;

repeat

Suma = Suma + PD(k) / SumInt;

k = k-1;

until Suma > 0.45;

end_repeat

PosMinMediana = k+1;

k = PosMediana;

Suma = 0;

repeat

Suma = Suma + PD(k)/SumInt;

k = k+1;

until Suma > 0.45;

end_repeat;

PosMaxMediana = k-1;

%It is defined the 90% of the power signal

MedAncho = FI(PosMaxMediana) - FI(PosMinMediana);

% PEAK POWER FREQUENCY FEATURE

max = PD(0); PosPico = 0;

for k = 0:1:5000

if max < PD(k);

then

max = PD(k);

PosPico = k;

end

end

FPico = FI(PosPico);

%HARMONIC INDEX

AreaEsp = 0;

for k = 0:1:5000

AreaEsp = AreaEsp + PD(k).*F(N)/5000;

end

HI =AreaEsp./ (F(N)*PD(PosPico));

%RELATIVE POWER CONTRIBUTION TO THE FIRST HARMONIC FEATURE

AreaEspSup = 0;

for k = PosMaxMediana:1:5000

AreaEspSup = AreaEspSup + PD(k).*F(N)/5000;

end

RPC = AreaEspSup.*RE;

%ANALYZED FEATURES OF GROUP A, PATIENT 8 AND HAND POSITION B

FEATURES = [FMediana, MedAncho, FPico, HI, RPC, RE];

save('FEATURES.txt', 'FEATURES', '-ascii');
